# Case report: Two individuals with *AEBP1*-related classical-like EDS: Further clinical characterisation and description of novel *AEBP1* variants

**DOI:** 10.3389/fgene.2023.1148224

**Published:** 2023-04-18

**Authors:** Chloe Angwin, Neeti Ghali, Fleur Stephanie van Dijk

**Affiliations:** ^1^ National Ehlers-Danlos Syndrome Service, London North West University Healthcare NHS Trust, London, United Kingdom; ^2^ Genetics and Genomics Division, Department of Metabolism, Digestion and Reproduction, Imperial College London, London, United Kingdom

**Keywords:** classical-like EDS type 2, Ehlers–Danlos syndrome, hair loss, AEBP1, connective tissue disease

## Abstract

**Introduction:** *AEBP1*-related classical-like EDS (clEDS type 2) is a rare type of Ehlers–Danlos syndrome (EDS) that was first reported in 2016. There are overlapping clinical features with *TNXB*-related classical-like EDS (or clEDS type 1), including skin hyperextensibility, joint hypermobility, and easy bruising. There are currently nine reported individuals with *AEBP1*-related clEDS type 2. This report confirms previous findings and provides additional clinical and molecular data on this group of individuals.

**Materials and methods:** Two individuals (P1 and P2), with features of a rare type of EDS, were clinically assessed in the London national EDS service and underwent genetic testing.

**Results:** Genetic testing in P1 revealed likely pathogenic *AEBP1* variants: c.821del:p. (Pro274Leufs*18) and c.2248T>C:p. (Trp750Arg). In P2 pathogenic *AEBP1* variants, c.1012G>T:p. (Glu338*) and c.1930C>T:p. (Arg644*) were identified.

**Discussion:** These two individuals increased the reported number of individuals with *AEBP1*-related clEDS to 11 (six females and five males). There are shared features with previously reported individuals, including hypermobility (11/11), skin hyperextensibility (11/11), presence of atrophic scarring (9/11), and easy bruising (10/11). In P1, a chronic right vertebral artery dissection, mild dilatation of the splenic artery, aberrant subclavian artery, and tortuous iliac arteries were observed at the age of 63 years. Cardiovascular disease has been reported, including mitral valve prolapse (4/11), peripheral arterial disease (1/11), and aortic root aneurysm requiring surgical intervention (1/11). Hair loss has been reported in 6/11 individuals (five females and one male), only one of which was documented to have a formal diagnosis of androgenetic alopecia, while other individuals were described as having thinning of hair, male pattern hair loss, or unspecified alopecia.

**Conclusion:** The clinical features of individuals with *AEBP1*-related EDS have not been fully elucidated yet. Hair loss is present in 6/11 individuals with *AEBP1*-related clEDS and appears to be a feature of this condition. This is the first time hair loss has been formally reported as a characteristic feature in a rare type of EDS. Cardiovascular surveillance seems warranted in this condition because 2/11 individuals have evidence of arterial aneurysm and/or dissection. Further descriptions of affected individuals are necessary to update diagnostic criteria and management guidelines.

## 1 Introduction

Classical-like EDS (clEDS) is a rare type of Ehlers–Danlos syndrome (EDS), termed type 1 or 2 according to the underlying genetic cause. Recessive variants in the gene *TNXB* encoding tenascin-X result in type 1 clEDS (Malfait et al., 2017), and recessive variants in the *AEBP1* gene encoding aortic carboxypeptidase-like protein (ACLP) result in type 2 clEDS (Ritelli et al., 2019).

Major diagnostic criteria for clEDS type 1 are as published in the 2017 International Classification of the Ehlers–Danlos syndromes: (i) skin hyperextensibility without atrophic scarring, (ii) generalised joint hypermobility, and (iii) easy bruising (Malfait et al., 2017). Minor diagnostic criteria for clEDS type 1 include (i) foot deformities, (ii) lower limb oedema, (iii) mild proximal and distal muscle weakness, (iv) axonal polyneuropathy, (v) atrophy of muscles in hands and feet, (vi) acrogeric hands, brachydactyly, clinodactyly, mallet finger, and (vii) vagina/uterus/rectal prolapse (Malfait et al., 2017). The minimal suggestive diagnostic criteria included all three major criteria and a family history suggestive of autosomal recessive transmission; confirmatory molecular testing is obligatory (Malfait et al., 2017).

Recessive variants in the *AEBP1* gene were first reported and associated with clEDS in two siblings (Alazami et al., 2016). (Alazami et al., 2016) (Alazami et al., 2016; Blackburn et al., 2018; Hebebrand et al., 2019; Ritelli et al., 2019; Syx et al., 2019) (Malfait et al., 2020) *AEBP1* encodes the aortic carboxypeptidase-like protein (ACLP), which is an extracellular matrix (ECM) protein identified in dermis, periosteum, vessel walls, and lung basement membrane with fundamental roles in embryogenesis and ECM repair and maintenance (Blackburn et al., 2018; Vishwanath et al., 2020). *AEBP1* variants, as a cause of clEDS type 2, were firmly established by Blackburn et al. in their 2018 paper, who described two individuals in addition to Alazami’s two individuals (Alazami et al., 2016; Blackburn et al., 2018). There are currently nine reported cases of clEDS as a result of variants in AEBP1 (Alazami et al., 2016; Blackburn et al., 2018; Hebebrand et al., 2019; Ritelli et al., 2019; Syx et al., 2019). Following these reports, distinctions have been drawn between clEDS type 1 and type 2, with type 2 including the presence of atrophic scarring and early-onset osteopenia (Malfait et al., 2020). This case summary presents two women with clEDS type 2 due to bi-allelic variants in *AEBP1*.

## 2 Case descriptions

### 2.1 Proband 1

At the time of writing, the proband (P1) was 65 years old. P1 was born at term after a normal pregnancy. At 18 months of age, bilateral dislocations of the hips were identified, requiring multiple surgeries continuing until the age of 10 years. As a child, P1 had significant joint hypermobility and required extractions for dental overcrowding. Joint dislocations after moderate trauma occurred throughout P1’s lifetime, with recurrent falls due to ankle instability and ongoing, progressive joint pain in her neck, back, and shoulders requiring regular analgesia. At age 55 and 60, P1 required a left hip replacement and a left knee replacement, respectively. P1 suffers from fatigue. From a young age, the proband bruised easily and severely after mild trauma, for example, large thigh haematomas from holding a baby on the proband’s lap. P1’s wound healing was normal and has reported heat intolerance due to reduced sweating, although this has not been further investigated.

P1 has had two children *via* caesarean section; both deliveries required blood transfusions due to haemorrhage, and one resulted in a large haematoma post-operatively. No other family members have a similar combination of clinical features. As a young child, hair loss was observed as a single occipital patch, which progressed to complete scalp hair loss after the birth of her first child at age 20, beginning with thinning over the vertex of the scalp and progressing to include the majority of the scalp.

**FIGURE 1:**
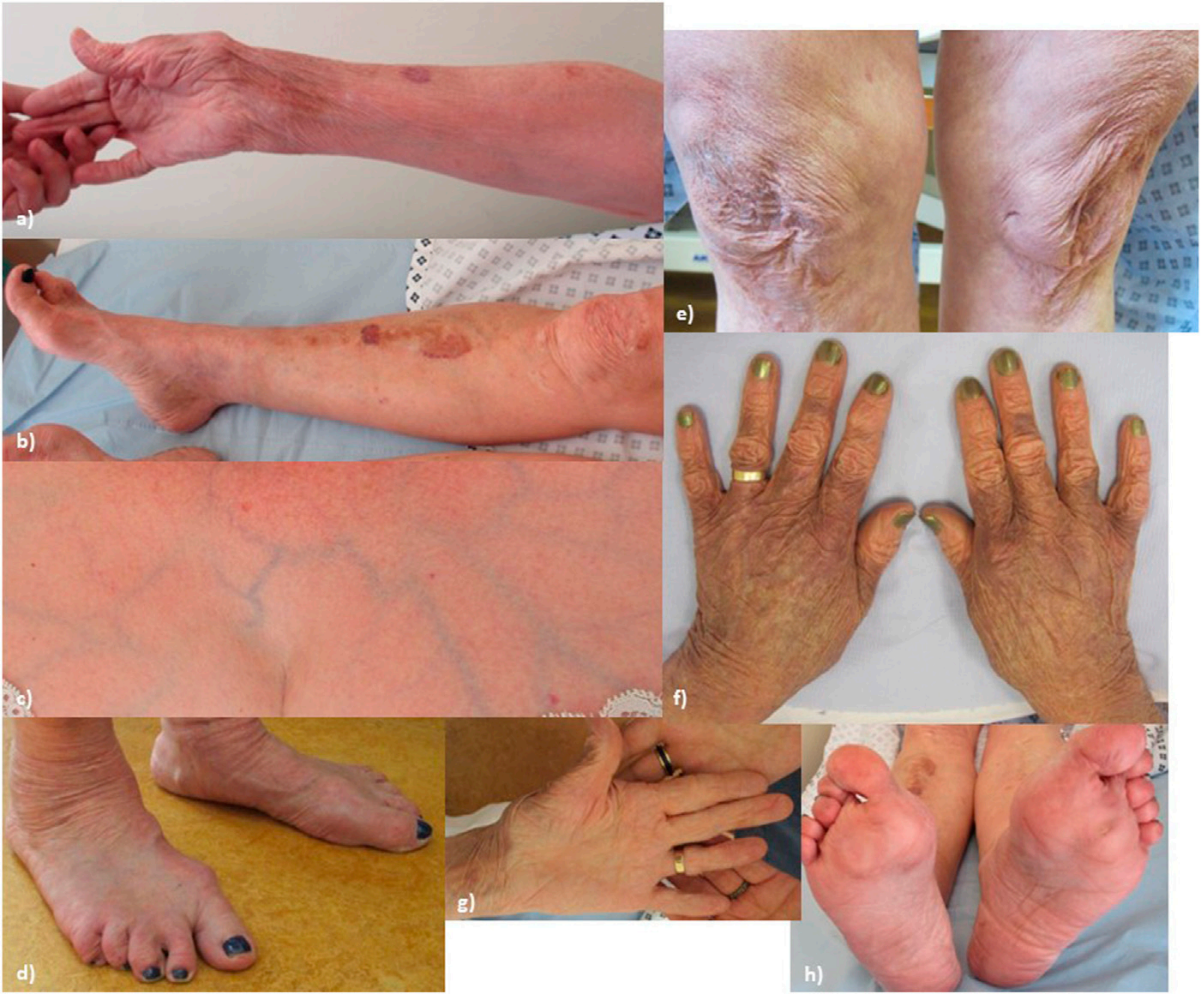
Proband 1: **(A)** increased palmar wrinkling, bruising, discolouration, and thinning of the skin over the forearms; **(B)** bruising and discolouration of shins; **(C)** skin over the chest is translucent with visible veins; **(D)** feet show pes planus with hammer toes and bilateral hallux valgus; **(E)** knees show mildly atrophic scars; **(F)** thin, discoloured skin over the dorsum of the hands; **(G)** increased palmar wrinkling and wasting of the thenar eminence; and **(H)** soles of the feet show an abnormal callus formation.

At the time of examination, age 62, (see Figure 1, patient’s consent has been gained for the use of all clinical photographs), P1 had a high arched palate (however, with no abnormally shaped teeth and no bifid uvula), with a scoliosis of the spine convex to the left. Earlobes were notched bilaterally. Pes planus was observed bilaterally, with hammer toes and bilateral hallux valgus. The skin was observed to be hyperextensible and lax, which was thin with translucent veins particularly over the chest. Bruising and discolouration of the arms was observed. Atrophic scars were observed over the knees and at the site of the left hip surgery. Thin scars observed over the forehead and at the site of the left knee surgery. P1’s Beighton score was 3/8. Hair loss was observed over the vertex and crown of the head. Body hair was also reduced including arms, legs, axillary, and pubic hair, while eyebrows and eyelashes were retained.

### 2.2 Investigations

A spine X-ray, at age 57, showed spondylotic changes, narrowing at C4-5 and C6-7 with severe degenerative change to the lumbar spine from L2 to L5. An echocardiogram conducted at age 54 was normal; however, at age 63, repetitive echocardiogram showed mild dilatation of the ascending aorta. A CT angiogram confirmed mild dilatation of the isthmus and ascending aorta (34 mm), as well as chronic right vertebral artery dissection, mild dilatation of the splenic artery, aberrant subclavian artery, and tortuous iliac arteries. P1 does not have cardiovascular risk factors (normotensive, BMI 22, and non-smoker).

**FIGURE 2:**
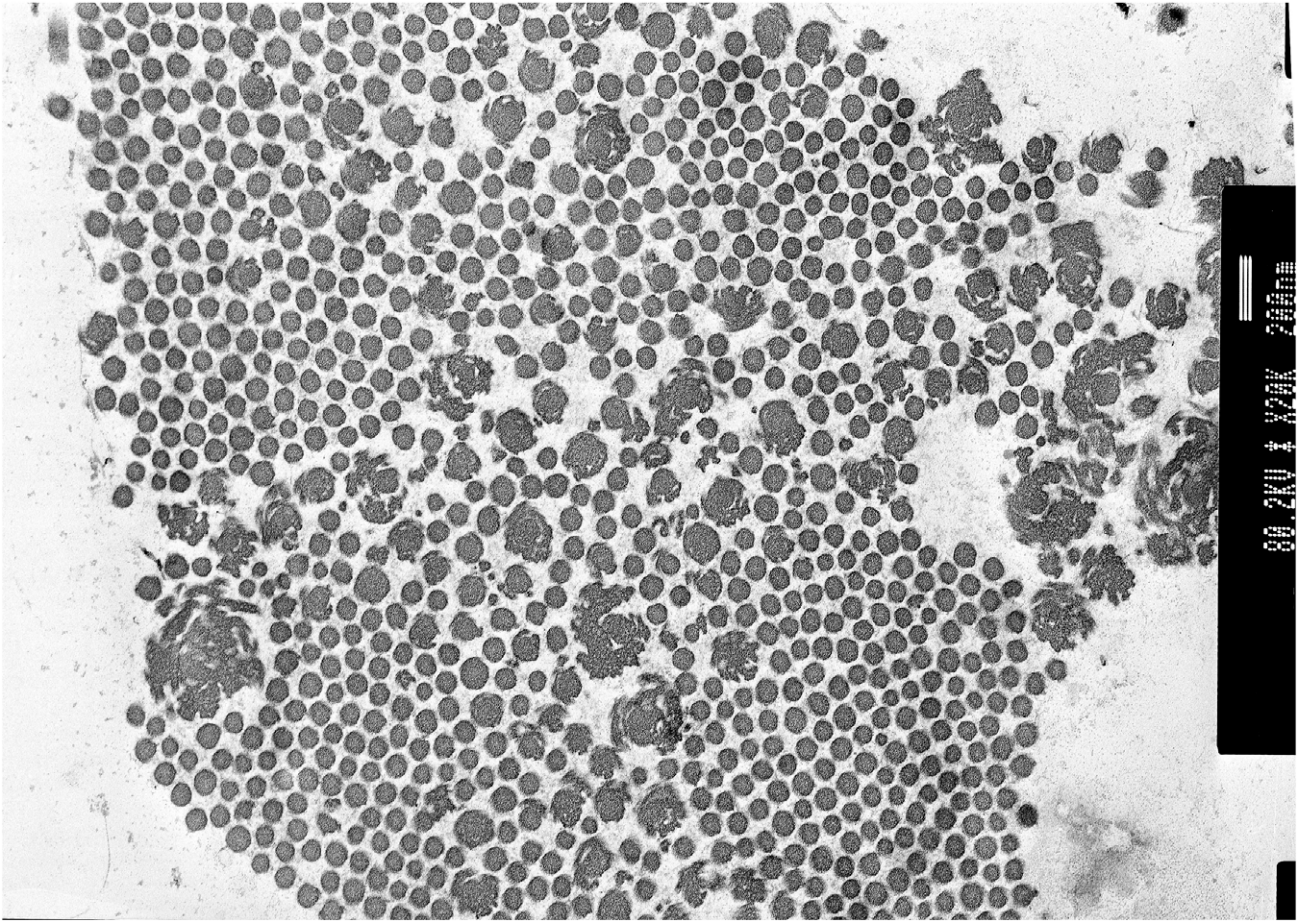
Proband 1: transmission electron microscopy (TEM) of the skin showing collagen flowers. TEM accelerating voltage at 80.2 kv and magnification ×20,000; scale bar shows 200 nm.

Transmission electron microscopy of skin biopsy taken from the inner upper arm showed abundant collagen flowers (see Figure 2).

Historical diagnostic DNA analysis including MLPA for classical and arthrochalasia EDS (*COL5A1, COL5A2, COL1A1,* and *COL1A2*), classical-like EDS type 1 (*TNXB*), and vascular EDS (*COL3A1* and *COL1A1*) showed no abnormalities. Therefore, at the age of 64, P1 was included in the United Kingdom national 100,000 Genomes Project, where likely pathogenic recessive compound heterozygous variants in *AEBP1* were identified *via* panel-based whole genome sequencing including all known genetic genes of rare EDS types, namely, c.821del; p. (Pro274fs) (PVS1, PM2, and PP4_str) and c.2248T>C p. (Trp750Arg) (PM2 and PP4_str), classified as ACMG class 4 (Richards et al., 2015; Caulfield et al., 2020).

### 2.3 Proband 2

At the time of writing, the proband (P2) was 41 years old. P2 was born prematurely at 35 weeks, with a low birth weight (2 kg and 25th centile) and was found to have bilateral talipes equinovarus, managed conservatively. P2 was hypermobile as a child with stretchy skin and had a left shoulder dislocation at the age of 1 year. P2 bruised easily and severely, including occasional haematomas which resolved spontaneously and required no drainage. P2’s skin is not particularly fragile. P2 has had two pregnancies with vaginal deliveries, where she sustained third-degree tears (obstetric anal sphincter injury), although these healed well. In the late twenties, P2 began to develop gradually worsening lumbar and hip pain, and fatigue with poor-quality sleep. Slow growing hair and generalised thinning of the hair on the scalp were also observed in P2’s 20s but were not treated. P2 has recently been diagnosed with high blood pressure, underactive thyroid, and pre-diabetes and has been prescribed clopidogrel, following a transient ischaemic attack. This was diagnosed following an episode of unilateral visual loss in the right eye, with no identified abnormalities on the echocardiogram or ultrasound of the neck. Investigative cardiac rhythm monitoring identified a short period of atrial flutter. An MRI of the head detected a small, old left temporal lacunar infarct, with no evidence of acute infarction. P2 is known to have osteopenia with reduced bone mineral density, along with some sites of osteoarthritis on further imaging of the joints.

**FIGURE 3:**
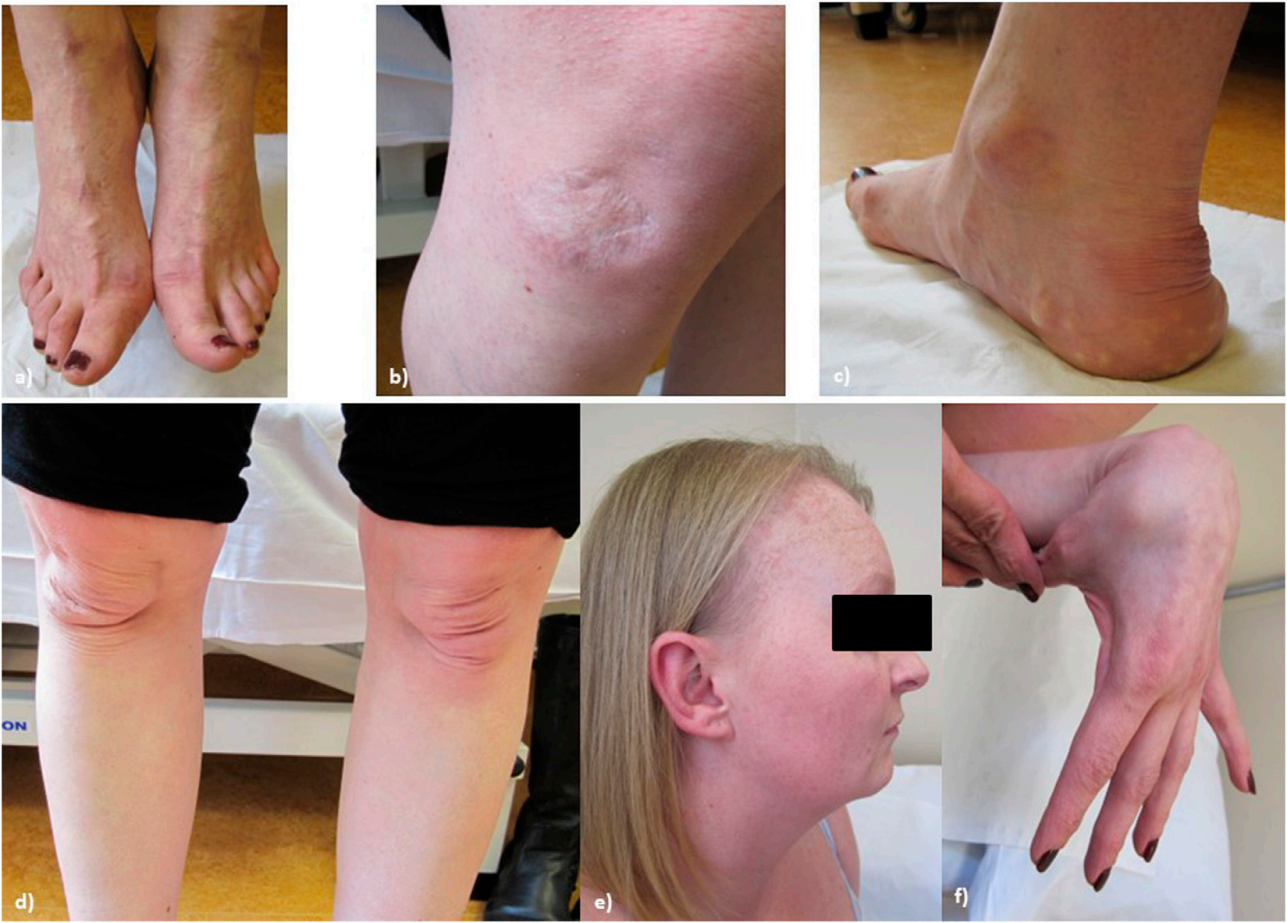
Proband 2: **(A)** bilateral hallux valgus, **(B)** atrophic scarring on the right knee, **(C)** piezogenic papules and pes planus, **(D)** bilateral patellar misalignment, **(E)** hair thinning around the frontal hairline and vertex, and **(F)** hypermobility in the hands.

On examination at age 34, (see Figure 3, patient’s consent has been gained for the use of all clinical photographs), P2 was observed to have no skeletal abnormalities. Earlobes were notched bilaterally. P2 had bilateral pes planus, with prominent piezogenic papules over both heels. The swas hyperextensible with thinning over the chest and papyraceous scarring over the right knee. Multiple subcutaneous spheroids were observed. Follicular keratosis of the skin of the neck and axillae was also observed. On examination, P2 had a single papyraceous scar on the knee, thin skin, and hair thinning over the scalp. There was generalised hypermobility (Beighton score 9/9) plus distal hypermobility of the small joints. P2’s hair was observed to be generally thin on the scalp; however, body hair was retained.

### 2.4 Investigations

**FIGURE 4:**
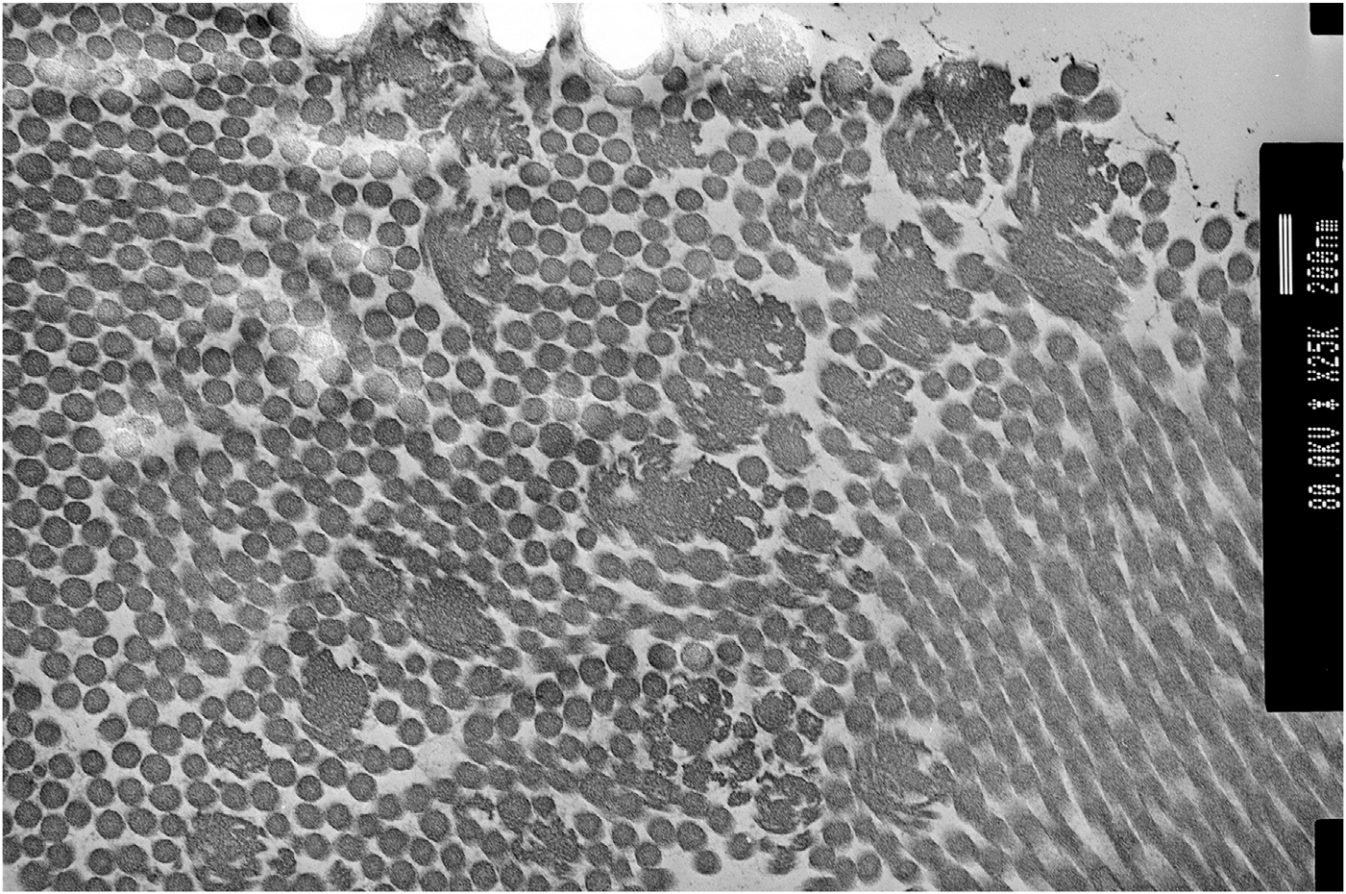
Proband 2: transmission electron microscopy (TEM) of the skin showing collagen flowers. TEM accelerating voltage at 80.2 kv and magnification ×20,000; scale bar shows 200 nm.

Genetic testing at the age of 36 for *COL1A1, COL3A1, COL5A1,* and *COL5A2* including MLPA showed no abnormalities. Transmission electron microscopy of skin biopsy taken from the inner, upper arm showed clumps of collagen fibres with large collagen flowers in the papillary dermis, with normal elastic fibres (see Figure 4). At the age of 38, P2 consented to participate in the 100K project (Caulfield et al., 2020); however, sequencing from the proband’s sample unfortunately failed. As such, sequencing of the United Kingdom EDS next-generation sequencing gene panel was arranged, which included *ADAMTS2, AEBP1, ALDH18A1, ATP6V0A2, ATP6V1A, ATP7A, B3GALT6, B4GALT7, BGN, C1R, C1S, CBS, CHST14, COL12A1, COL1A1, COL1A2, COL3A1, COL5A1, COL5A2, COL6A1, COL6A2, COL6A3, DSE, EFEMP2, ELN, FBLN5, FBN1, FBN2, FKBP14, GORAB, LOX, LTBP4, PLOD1, PRDM5, PYCR1, RIN2, ROBO3, SKI, SLC39A13, SMAD2, SMAD3, TGFB2, TGFB3, TGFBR1, TGFBR2, TNXB,* and *ZNF469*. Recessive variants were identified in *AEBP1* (NM_001129.4) c.1012G>T; p. (Glu338*) (PM2, PVS1, and PM3_sup) and c.1930C>T, p. (Arg644*) (PM2, PVS1, and PM3_sup), classified as ACMG class 5 (Richards et al., 2015).

## 3 Discussion

Recently, clEDS has been stratified into two types (1 and 2), according to the underlying genetic cause (recessive deleterious variants in *TNXB* or *AEBP1*). ClEDS type 1 was originally defined to differentiate classical EDS (cEDS), which is associated with atrophic scarring, from clEDS where scarring tends to be normal. Although there are some clinical similarities between the two clEDS types, including joint hypermobility, easy bruising, hyperextensible skin, and foot abnormalities, clEDS type 2 is reported to have additional features, including atrophic scarring and early-onset osteopenia (Malfait et al., 2020). The two individuals presented here share similar clinical features to each other, including joint hypermobility, fatigue, severe and easy bruising, hyperextensible skin with mild atrophic scarring to normal scarring, and generalised hair thinning over the scalp. Both individuals have collagen flowers on TEM, which has previously been observed in this cohort of patients (Blackburn et al., 2018).

ClEDS type 2 appears to be rare, with only nine reported individuals (Alazami et al., 2016; Blackburn et al., 2018; Hebebrand et al., 2019; Ritelli et al., 2019; Syx et al., 2019), and 11 (six females and five males), including the two cases presented here, with the mean age being 38 (range 12–65) (see table 1). There are shared features with other previously reported individuals, particularly hypermobility (11/11), skin hyperextensibility (11/11), atrophic scarring (reported in 9/11), and easy bruising (10/11) (Blackburn et al., 2018; Hebebrand et al., 2019; Ritelli et al., 2019). Cardiovascular disease has been reported as an aortic root aneurysm, requiring surgical intervention in one (1/11) person at age 36, without known cardiovascular risk factors. This individual also had a bowel rupture at a young age and suffered with significant scarring and incisional hernias (Blackburn et al., 2018). Other cardiovascular complications include mitral valve prolapse (4/11) that was discovered at ages 33, 35, 39, and 58, and peripheral arterial disease (1/11) diagnosed at age 53 (Blackburn et al., 2018; Ritelli et al., 2019). In P1, the eldest patient reported, a chronic right vertebral artery dissection, mild dilatation of the splenic artery, aberrant subclavian artery, and tortuous iliac arteries were observed at age 63 (Ritelli et al., 2019) (Hebebrand et al., 2019; Syx et al., 2019) (Blackburn et al., 2018). Osteopenia was identified in 6/11 patients including proband 2 (aged 12, 24, 33, 35, 53, and 41 years at the time of reporting) from this paper, three of whom had had fractures (Blackburn et al., 2018; Ritelli et al., 2019).

**TABLE 1:** Summary table of currently reported cases (Alazami et al., 2016; Blackburn et al., 2018; Hebebrand et al., 2019; Ritelli et al., 2019; Syx et al., 2019); F: female; M: male.

Paper	Alazami	Alazami	Blackburn	Blackburn	Hebebrand	Hebebrand	Ritelli	Syx	Syx	This paper	This paper
Case number	Family 1 IV:4	Family 1 IV:6	A-II:1	B-II:1	D-II:1	D-II:2	P1	P1	P2	P1	P2
Age in the report	12	24	35	33	39	38	53	58	21	65	41
Gender	F	M	M	M	F	M	F	M	F	F	F
*AEBP1* variant 1	c. [1630 + 1G>A]	c. [1630 + 1G>A]	c. [1470del]	c. [1320 1326del];	c. [917dup]	c. [917dup]	c. [1925T>C]	c. [362dupA]	c. [443dupA]	c. [821del]	c.1012G>T;
	p. (Val537Leufs*31)	p. (Val537Leufs*31)	p. (Asn490_Met495delins (40))	p. (1320_1326del)	p. (Tyr306*)	p. (Tyr306*)	p. (Leu642Pro)	p. (Glu122Glyfs*16)	p. (Ala149Glyfs*57)	p. (Pro274Leufs*18)	p. (Glu338*)
*AEBP1* variant 2	c. [1630 + 1G>A]	c. [1630 + 1G>A]	c. [1743C>A]	c. [1320 1326del];	c. [917dup]	c. [917dup]	c. [1925T>C];	c. [362dupA]	c. [1149 1150+2del]	c. [2248T>C]	c. [1930C>T]
	p. (Val537Leufs*31)	p. (Val537Leufs*31)	p. (Cys581*)	p. (1320_1326del)	p. (Tyr306*)	p. (Tyr306*)	p. (Leu642Pro)	p. (Glu122Glyfs*16)	p. loss of donor splice site of exon 9	p. (Trp750Arg)	p. (Arg644*)
Skin hyperextensibility	+	+	+	+	+	+	+	+	+	+	+
Scarring	Atrophic, keloid, and hyperpigmentation	Unknown	Atrophic	Atrophic and hyperpigmentation	+	+	Atrophic	Atrophic	No scarring	Atrophic	Atrophic
Easy bruising	+	Unknown	+	+	+	+	+	+	+	+	+
Joint hypermobility	+	+	+	+	+	+	+	+	+	+	+
Beighton score	8/9	Unknown	8/9	8/9	6/9	2/9	5/9	Unknown	9/9	3/9	9/9
Joint dislocations	+	Unknown	+	+	+	+	+	+	+	+	+
Osteopenia	+	+	+	+	Unknown	Unknown	+	Unknown	-	-	+
Mitral valve prolapse	-	-	+	+	+	-	-	+	-	-	-
Aortic dilatation	-	-	-	+	-	-	-	-	-	+	-
Hair thinning	Unknown	Unknown	Unknown	Unknown	Alopecia	Unknown	Alopecia	Thin, frizzled hair with partial alopecia	Thinning of hair	Hair loss on the scalp with reduced body hair	Thinning of hair
Other				Aortic root aneurysm and bowel rupture			Peripheral vascular disease			Vertebral artery dissection	

### 3.1 *AEBP1*

The *AEBP1* gene encodes two protein isoforms, AEBP1 and ACLP (Majdalawieh et al., 2020). AEBP1 is a transcriptional repressor of anti-inflammatory and apoptotic genes in the nucleus and can also alter cellular signalling *via* a protein–protein interaction in the cytosol (Majdalawieh et al., 2020). However, recessive *AEBP1* variants, causative of clEDS type 2, result in the disruption of ACLP (Blackburn et al., 2018; Vishwanath et al., 2020). The ACLP protein is extracellularly secreted and associated with the ECM; it is particularly expressed in the dermis, lung basement membrane, medial layer of blood vessels, and the periosteum (Blackburn et al., 2018). Post embryogenesis, ACLP has been found to be expressed during wound healing and after vessel injury (Vishwanath et al., 2020). ACLP binds with collagens in the ECM and has been found to reduce the collagen fibre diameter and increase toughness (Vishwanath et al., 2020). ACLP is also involved in intracellular signalling *via* the TGFβ pathway (Tumelty et al., 2014) and is involved in WNT/β-catenin pathway signalling through WNT3A (Teratani et al., 2018).

### 3.2 *AEBP1*-related classical-like EDS and hair loss

Hair loss was reported in 6/11 individuals (five females and one male), only one of which was documented to have a formal diagnosis of androgenetic alopecia (Ritelli et al., 2019), while other individuals were described as having thinning of hair, male pattern hair loss, or unspecified alopecia (Hebebrand et al., 2019; Syx et al., 2019). In other reports, hair loss is not specifically mentioned and may have been presented as a feature and not reported on. One individual reported impaired temperature sensation, but none reported reduced sweating (Blackburn et al., 2018).

Intriguingly, 6/11 individuals with *AEBP1*-related classical-like EDS experienced hair thinning and loss resembling androgenetic alopecia. So far, hair loss has not been reported in other EDS types as a typical clinical feature; however, it has been reported to be occurring inconsistently in some women with vascular EDS (Byers et al., 2017). Androgenetic alopecia, a progressive type of hair loss which affects men and women, is characterised by gradual thinning of the hair particularly over the frontal hairline, temples, and vertex, which can progress to complete hair loss over the scalp (Lolli et al., 2017). The mechanism behind these changes is not clearly understood, but there is a clear link with increased follicular sensitivity to androgens (Lolli et al., 2017). ECM changes in androgenetic alopecia have been observed but are not fully understood and include the altered deposition of elastin fibres, changes to the follicle sheath, and disruption of the basal lamina (Rushton et al., 2021). Some studies have suggested that the WNT/β-catenin pathway is the main pathway involved in the progression of androgenetic alopecia, given that androgen receptors interact with β-catenin in an androgen-dependent manner (Chesire & Isaacs, 2002; Lolli et al., 2017; Doolan et al., 2021) These findings suggest that androgens deregulate normal hair follicle differentiation *via* inhibition of the WNT pathway, which has been found to maintain the hair follicle (Kishimoto et al., 2000; Leirós et al., 2012; Lolli et al., 2017).

In individuals with pathogenic *AEBP1* variants, it is possible that WNT signalling is disrupted, resulting in this specific phenotype with androgenetic alopecia. However, given the rarity of *AEBP1*-related clEDS, there are currently no published data to support this theory.

### 3.3 Management recommendations

There are currently no management recommendations for type 2 clEDS published in the literature. Generalised tissue fragility, including vascular fragility, has been reported in several rare EDS types, including type 1 clEDS and vEDS, where vascular fragility is a major clinical feature (Malfait et al., 2017; van Dijk et al., 2022). Although there is a lack of natural history data on individuals with pathogenic *AEBP1* variants, there are reports of vascular complications in a high number of reported individuals and there is evidence that ACLP is a component of the arterial tunica media (Blackburn et al., 2018). This group may therefore be at risk of cardiovascular events including arterial dissections, as observed in proband 1 of this paper. We would, therefore, recommend that individuals with clEDS type 2 undergo cardiovascular investigation at diagnosis and have ongoing surveillance depending on age, symptoms, results of initial cardiovascular investigations, and considering surveillances in other rare EDS types until data on more individuals with this diagnosis become available.

As 6/11 patients had osteopenia, three of whom sustained fractures, a DEXA scan and bone markers for osteoporosis in blood and/or urine during diagnosis may be advisable in order to initiate appropriate management when necessary.

Hair loss can carry a significant psychological burden (Hunt & McHale, 2005), and patients who are experiencing distress must be referred for specialist support and consideration of management options. Both individuals reported here have been referred for evaluation by a specialist and consideration of management options.

## 4 Summary

This report demonstrates and expands the phenotypic spectrum of bi-allelic pathogenic *AEBP1* variants, resulting in clEDS type 2. ClEDS type 2 is an important differential in patients with joint hypermobility, skin hyperextensibility, easy bruising, hair loss, and osteopenia. Hair loss appears to be an important clinical feature in this EDS type that has not been reported in the literature as a consistent clinical feature in other types of EDS. We have made recommendations based on the current literature; however, there are currently no consensus guidelines on management.

## Data Availability

The datasets for this article are not publicly available due to concerns regarding participant/patient anonymity. Requests to access the datasets should be directed to the corresponding author.
